# Effects of prolonged exposure to ELF-EMF on HERVs expression in human melanoma cells

**DOI:** 10.22099/mbrc.2022.42754.1706

**Published:** 2022-06

**Authors:** Abbas Karimi, Farzaneh Ghadiri-Moghaddam, Masoumeh Valipour, Yahya Yahyavi

**Affiliations:** 1 Biotechnology Research Center, Tabriz University of Medical Sciences, Tabriz, Iran; 2 Department of Molecular Medicine, Faculty of Advanced Medical Sciences, Tabriz University of Medical Sciences, Tabriz, Iran; 3 Department of Biology, Faculty of Science, Azarbaijan Shahid Madani University, Tabriz, Iran

**Keywords:** Extremely low-frequency electromagnetic fields (ELF-EMFs), Prolonged exposure, Human Endogenous Retrovirus, Melanoma

## Abstract

The Human endogenous retroviruses (HERVs) are ancient remnants of exogenous retroviral infections. Their abnormal activation is associated with several diseases, such as cancer and autoimmunity. Epigenetic and environmental factors are probably playing essential roles in the expression of these elements. This study aimed to examine the 96-hour effects of ELF-EMF on HERV-H, K, and W expression in human melanoma cells. SK-MEL-37 cells (the human skin malignant melanoma) were continuously exposed to ELF-EMF (50 Hz) at 1.5 and 3 mT intensity for 96 hours. Following mRNA extraction, the expression level of HERV-H, K, and W was assessed by qPCR. According to our results, exposure to ELF-EMF intensities for 96 hours could significantly downregulate HERV-H, K, and W env gene expression (P<0.001). Our obtained data suggest that low intensity and long-term exposure to ELF-EMF may pave using this type of radiation as a novel therapeutic approach by neutralizing the HERVs upregulated expression in melanoma cells.

## INTRODUCTION

Human endogenous retroviruses (HERVs) are long terminal repeats containing (LTR) retro-elements, that originated from exogenous retroviruses, constitute approximately 8% of the human genome [1, 2]. A full-length HERV provirus composed of two LTRs that flank four major open reading frames (ORFs), including *gag*, *pro, pol, *and *env *genes [3]. HERV LTRs in some genomic locations can act as transcriptional promoters/enhancers or repressors and may alter the expression pattern of adjacent genes [4]. Insertional mutagenesis mediated by HERVs elements and functional retroviral proteins exhibits well-established pathogenic potential. They may abnormally regulate the expression of specific transcripts by insertion of HERVs promoter sequences [5, 6]. Some internal and external factors such as steroid hormones, cytokines, viral and microbial infections, chemical substances like heavy metals, and physical inducing agents can affect the activation of HERVs elements [7-9]. Extremely low frequency (ELF) electric and magnetic fields (EMF) are non-ionizing radiation with a range of radiation frequencies from 0 to 3 kHz. Exposure to ELF-EMF results from various sources, such as proximity to electric power lines, wiring, and electrical household appliances [10]. In the list of agents classified by the International Agency for Research on Cancer (IARC) monograph, ELF-EMF is in Group 2B agents as possibly carcinogenic [11]. This study aims to evaluate the effect of the ELF-EMF on HERV-K-W-H *env *genes induction in the SK-MEL-37 melanoma cell line.

## MATERIALS AND METHODS

Human malignant melanoma cells (SK-MEL-37 cells) were cultured in RPMI 1640 medium (Gibco™, USA) with 10% (v/v) heat-inactivated fetal bovine serum (Gibco™, USA) and 1% (w/v) Pen/Strep (100 U/ml penicillin, and 100 μg/ml streptomycin) (Gibco™, USA). The cells were grown under a humidified atmosphere of 95% air and 5% CO_2_ at 37°C.

To study the effects of 96-hour low intensities exposure to ELF-EMF on melanoma cells, we generated an ELF-EMF generator in an incubator. The exposure system consisted of the solenoid with 200 turn copper wire that was double-wrapped to obtain active and sham configuration. In the sham configuration, MF was considered zero. The coils were placed in a stainless iron structure in a 5% CO_2_ incubator. The electromagnetic apparatus generated o 0.5-mT to 3 mT, 50-Hz sinusoidal magnetic field. The EMF strength was measured by a Teslameter. Temperature, CO_2,_ and humidity were adjusted for cell culture conditions and controlled over the experiment duration. For exposure purposes (0 to 3 mT), 310^5^ cells were seeded on T-25 cell culture flask (25 cm^2^) and placed on the center of the incubator between the coil systems to provide a uniform MF during the exposure period.

At the end of the exposure time, RNA was extracted from the cells and converted into cDNA as described [9]. Using primer specific for HERV-H, HERV-K, HERV-W *env* gene, and *GAPDH* (Table 1), the real-time PCR was carried out in the Mic qPCR Cycler instrument (BioMolecular Systems, Australia). The gene expression ratio was normalized, according to the 2^–ΔΔCT ^method. All data are shown as mean ± SD of three independent samples. The Mann–Whitney U test was used to compare the difference between two independent samples. A P-value smaller than 0.05 was considered significant.

**Table 1:** A list of specific primers used for amplification of selected HERVs *env* gene

**Gene**	**Accession number**	**Sequences (5´->3´)**	**Tm (°C)**	**Product length (bp)**
*GAPDH *	NM_002046.7	F: AATCCCATCACCATCTTCCA R: TGGACTCCACGACGTACTCA	59	82
HERV-K *env*	NC_000010.11	F: TAATTTACCCGTGGCCTGAG R: GCAGTCCAAAATTGGTTGGT	58	101
HERV-W *env*	NM_014590.4	F: TGCTAACCGCTGAAAGAGGG R: CGAAGCTCCTCTGCTCTACG	60	136
HERV-H *env*	AJ289711.1	F: TGGCCGCTCCTTTATGTATC R: TAGTTGGGCTTTGGAGATGG	58	105

In this study, we had not any experiments on human and animal samples so we did not need informed consent. This study was approved by the Tabriz University of Medical Sciences Human Research Ethics Committee in Iran (Ref no: IR.TBZMED.REC.1397.973).

## RESULTS AND DISCUSSION

**Figure 1:**
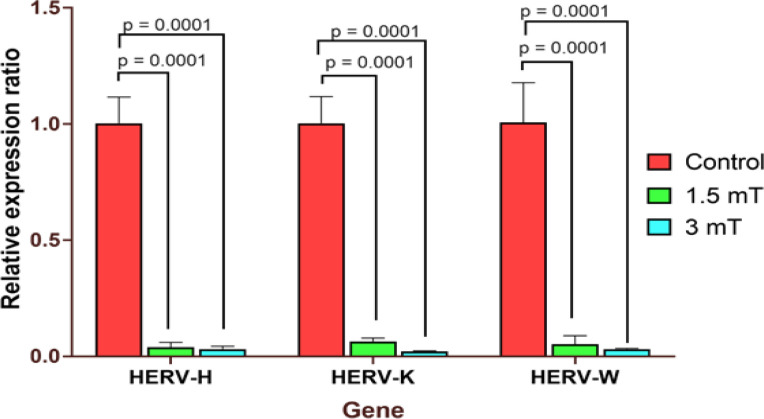
Effect of exposure to ELF-EMF on HERVs expression in SK-MEL-37 cells. To evaluate the effects of long-term exposure to ELF-EMF on HERVs expression, the SK-MEL-37 cells were exposed to ELF-EMF at 0 (control), 1.5 and 3 mT for 96 hrs. Then expression levels of HERV-H, HERV-K, and HERV-W in the env gene were measured. The results showed significant downregulation in all exposed groups (P<0.001)

In this study, we assessed 96-hour exposure of SK-MEL-37 cells to ELF-EMF at 0, 1.5, and 3 mT intensities. The effects of prolonged ELF-EMF exposure on studied HERVs mRNA expression are reported in Fig. 1. According to the results, the HERV-H *env* gene had a significant downregulated level at 1.5 and 3 mT intensity compared with unexposed cells (P<0.001). Besides, exposure to 1.5 and 3 mT ELF-EMF waves significantly downregulated the expression of HERV-K (P<0.001). Regarding the HERV-W, both intensities also significantly downregulated *env* expression (P<0.001).

HERVs are genetic parasites and have high expression levels in some autoimmune diseases and cancers. HERV Env proteins induce abnormal cell-cell fusion and possibly contributing to tumor development and metastasizing processes [12]. Recent evidence suggests that these elements may be used as a biomarker for diagnostic and therapeutic targets. For instance, Attention-deficit/hyperactivity disorder (ADHD) patients treated with methylphenidate (MPH) have shown decreased HERV-H expression, which was associated with improved disease [13]. In multiple sclerosis patients, a decreased HERV-W expression level in patients under interferon-beta therapy has been reported [14]. Based on previous studies, ELF-EFM was expected to induce HERVs expression; but we found that this exposure can ameliorate HERVs destructive effects in melanoma cells. However, the exact mechanism of such downregulation in HERVs by EMF exposure is not known; more likely, the methylation of CpG island in LTR promoter regions may reduce the expression of HERV genes [15].

Moreover, 50 Hz ELF-EMF exposure with low intensities may alter DNA methylation patterns in some genes [16]. Besides, ELF-EMF exposure affects mRNA expression levels of some genes involved in antioxidant pathways [17]. This phenomenon can be extended in the context of HERVs elements. Similar to our results, Del Re *et al*., [18] showed ELF-PMF (pulsed magnetic field) decreases LINE-1 retrotransposition activity in neuroblastoma cells. Another study with this research group indicated a decrease in DNA methylation of different CpG islands following exposure to a combination of oxidative stress with ELF-PMF (PMF; 50 Hz, 1 mT) [19]. According to the structure of our incubator, studying cell viability and cell growth of different study groups at the same time and similar conditions was not possible. However, we assessed the cell viability of control and one of the treated groups in unsynchronized way that was not significant (data not shown). 

This was the first study reporting the ELF-EMF's prolonged effect on HERVs gene expression, so a more in-depth insight into the DNA methylation status of HERVs activation is needed following exposure to ELF-EMF on tumoral cells. Further studies are required to evaluate underlying potential mechanisms around ELF-EMF with more emphasis on therapeutic targets on skin cancer. 

## Conflict of Interest

The authors declare no conflict of interest.
